# Systemic Evaluation on the Pharmacokinetics of Platinum-Based Anticancer Drugs From Animal to Cell Level: Based on Total Platinum and Intact Drugs

**DOI:** 10.3389/fphar.2019.01485

**Published:** 2020-01-08

**Authors:** Zhiying Qin, Guanghui Ren, Jinjie Yuan, Huili Chen, Yang Lu, Ning Li, Yongjie Zhang, Xijing Chen, Di Zhao

**Affiliations:** ^1^ Clinical Pharmacokinetics Laboratory, China Pharmaceutical University, Nanjing, China; ^2^ School of Engineering & Applied Science, Yale University, New Haven, CT, United States; ^3^ National Experimental Teaching Demonstration Center of Pharmacy, China Pharmaceutical University, Nanjing, China

**Keywords:** cisplatin, carboplatin, oxaliplatin, total platinum, pharmacokinetics

## Abstract

Cisplatin, carboplatin, and oxaliplatin are the common platinum-based anticancer drugs widely used in the chemotherapeutic treatment of solid tumors in clinic. However, the comprehensive pharmacokinetics of platinum-based anticancer drugs has not been fully understood yet. This leads to many limitations for the further studies on their pharmacology and toxicology. In this study, we conduct a systemic evaluation on the pharmacokinetics of three platinum analogues at animal and cell levels, with quantification of both total platinum and intact drugs. A detailed animal study to address and compare the different pharmacokinetic behaviors of three platinum analogues has been conducted in three biological matrices: blood, plasma, and ultrafiltrate plasma. Carboplatin showed an obviously different pharmacokinetic characteristic from cisplatin and oxaliplatin. On the one hand, carboplatin has the highest proportion of Pt distribution in ultrafiltrate plasma. On the other hand, carboplatin has the highest intact drug exposure and longest intact drug elimination time in blood, plasma, and ultrafiltrate plasma, which may explain its high hematotoxicity. Additionally, the cellular and subcellular pharmacokinetics of oxaliplatin in two colon cancer HCT-116/LOVO cell lines has been elucidated for the first time. The biotransformation of intact oxaliplatin in cells was rapid with a fast elimination, however, the generated platinum-containing metabolites still exist within cells. The distribution of total platinum in the cytosol is higher than in the mitochondria, followed by the nucleus. Enrichment of platinum in mitochondria may affect the respiratory chain or energy metabolism, and further lead to cell apoptosis, which may indicate mitochondria as another potential target for efficacy and toxicity of oxaliplatin.

## Introduction

Platinum analogues have been employed as drug agents to treat certain types of solid tumors since the advent of cisplatin in 1978(1). Cisplatin, carboplatin, and oxaliplatin are respectively the first, second, and third generation classic platinum-based anticancer drugs that are now frequently used in the chemotherapeutic treatment of malignancies. It is generally believed that all these platinum complexes act on DNA in the nucleus, inhibiting DNA replication and thus exerting cytotoxic effects (Dasari and Tchounwou, 2014; Corte-Rodriguez et al., 2015). Although the mechanisms of these platinum-based anticancer drugs are similar, their primary indications and side effects are different. For example, cisplatin has a specific efficacy in the treatment of testicular cancer, and head and neck cancer in clinic. Carboplatin is mainly used in clinical treatment of non-small cell lung cancer. Preferably, oxaliplatin exhibits the best therapeutic effect on colon cancer (Rabik and Eileen, 2007; Chen and Lin et al., 2017; Lee et al., 2018). At the same time, in terms of toxicity, the main side effect of cisplatin is nephrotoxicity; bone marrow toxicity is more serious for carboplatin; and oxaliplatin mainly manifests as peripheral neurotoxicity (Knox et al., 1986; Chovanec et al., 2017; Griffith et al., 2017; Marmiroli et al., 2017; Milic et al., 2019). However, the pharmacology and toxicology of these platinum-based anticancer drugs have not been fully elucidated yet (Hanada et al., 2008). Although closely related to a drug’s efficacy and toxicity (Hanada et al., 2010; Rizk et al., 2017), the systematic comparison of pharmacokinetics of these three platinum analogues and the substance basis for their efficacy is not fully clear. Due to the potent electrophilicity of platinum-based anticancer drugs, a series of biotransformation reactions occur *in vivo*7,15. In addition to affinity with DNA, the platinum analogues can perform spontaneous chemical reactions and irreversibly bound to proteins or other low molecular weight compounds (Esteban-Fernandez et al., 2007; Pinato et al., 2013; Wenzel and Casini, 2017). Therefore, the intact platinum-based anticancer drugs can be biotransformed into a number of other platinum-containing metabolites *in vivo*. Some of the platinum-containing hydrolyzation products are effective while others are not or toxic (Michalke, 2010). Moreover, the active substances that actually exert antitumor effects are not yet clear. Although distinguished systemic pharmacokinetics of cisplatin, carboplatin, and oxaliplatin has already been investigated extensively and reviewed numerously (O’Dwyer et al., 2000), most of the previous reports are only based on quantification of total platinum with a lack of determination of intact drugs. In addition, several studies on the pharmacokinetics of platinum analogues with quantification of intact drugs have also been reported (Xie et al., 2017; Qin et al., 2018; Ren et al., 2018). However, there is still lack of simultaneous determination of both total Pt and intact drugs to characterize the pharmacokinetics of platinum-based drugs, which can help understand the behaviors of intact platinum-based drug and its biotransformation better. In clinic, distribution in blood is the first step of platinum-based anticancer drugs in the body, which will inevitably affect its efficacy and toxicity. Therefore, exploring the pharmacokinetics and distribution of total platinum and intact drugs in blood has great values for understanding the behaviors of platinum-based drugs *in vivo*.

On the other hand, platinum-based anticancer drugs interact with targets localized in cells, for which the intracellular distribution of drugs and concentrations around the target are critical to efficacy (Zhou et al., 2011). Although cellular uptake characteristics of platinum-based drugs has already been investigated (Dirk et al., 2012), most of the previous reports are only based on quantification of total platinum without characterization of intact drugs in cells. In addition, the subcellular distribution of platinum-based drugs has already been investigated by (Esteban-Fernandez et al., 2008), however, they focused on the subcellular distribution of platinum-based drugs in normal tissue and cells but not the target cancer cells. Due to the importance of cellular pharmacokinetics or subcellular distribution of platinum analogue in understanding its pharmacology and toxicity, there is an urgent need to quantitatively evaluate the intracellular concentration and subcellular distribution of platinum-based anticancer drugs to clarify the cellular pharmacokinetics of platinum analogues in target cells.

The aim of the study was to systematically evaluate the pharmacokinetics and distribution of classic platinum-based anticancer drugs from animal to cell model, with quantification of total platinum by inductively coupled plasma mass spectrometry (ICP-MS) method and intact drug by ultra performance liquid chromatography/tandem mass spectrometry (UPLC-MS/MS). For the first time, the total platinum and intact drug distribution of cisplatin, carboplatin, and oxaliplatin in rat blood, plasma, and plasma ultrafiltrate were investigated comprehensively. Furthermore, we chose to investigate the pharmacokinetic behaviors and subcellular distribution of third-generation platinum-based drug oxaliplatin in colon cancer target cells, because of its most prominent targeted indication compared to cisplatin and carboplatin. In the paper, a systematic and multi-view pharmacokinetic study for platinum-based anticancer drugs will be presented.

## Materials and Methods

### Chemicals and Reagents

**Figure 1:**
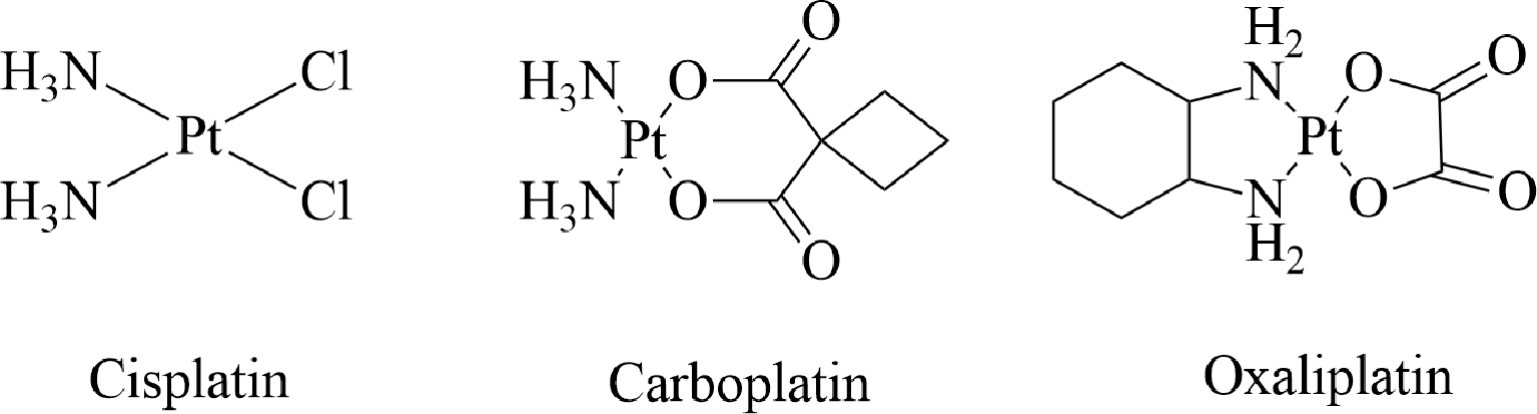
Chemical structures of cisplatin, carboplatin and oxaliplatin.

Cisplatin, carboplatin, and oxaliplatin (**Figure 1**) were all purchased from Raw Material Medicine reagent co. LTD (Nanjing, China), with a purity of 99%. Carboplatin-d4 (internal standard) was purchased from Toronto Research Chemical (Toronto, Canada), with a purity of 98%. Rhodium Standard and Platinum Standard for ICP were both purchased from SIGMA-ALDRICH (St Louis, MO, USA). Triton™ X-100 was also purchased from SIGMA-ALDRICH (St Louis, MO, USA). Nitric acid was purchased from Sinopharm Chemical Reagent Co., Ltd (Shanghai, China). Acetonitrile of HPLC-grade was all purchased from TEDIA (Fairfield, CT, USA). Ultra-pure water was obtained from a Milli-Q^®^ (Millipore) water purifying system (Millipore Corp, Bedford, MA, USA). Ammonium acetate was purchased from Xilong Scientific (Shanghai, China). McCoy’s 5a Medium, F-12K medium, Fetal bovine serum (FBS), Hank’s Balanced Salt Solution (HBSS), bicinchoninic acid (BCA) protein assay kit, and 0.25% trypsin were purchased from KeyGEN BioTECH (Nanjing, China).

### Animals and Cell Lines

Sprague-Dawley (SD) rats (male, 200–220 g) are all purchased from Qinglongshan Experimental Animal Company Ltd. (Jiangsu, China).

Human colon cancer HCT-116 and LOVO cell line was purchased from cell bank of Shanghai Institute of Biochemistry and Cell Biology (Shanghai, China). HCT-116 cells and LOVO cells were maintained as monolayer cultures respectively in McCoy’s 5a medium and F-12K medium, supplemented with 10% fetal bovine serum, 1% penicillin and streptomycin, in a water-saturated atmosphere (95% air/5% CO2) at 37°C. Cells were used for studying cellular pharmacokinetics of oxaliplatin.

### Instrumentation

#### Sample Treatment and Analysis for Total Pt Determination by ICP-MS

The biological samples were pretreatmented by adding 60-fold volume of ICP-MS diluent, which is an aqueous solution containing 2% nitric acid and 0.5% Triton. After vortex for 30 s, the samples were placed quietly for few minutes to obtain a clear supernatant for ICP-MS analysis of total platinum.

Platinum determination was performed with a NexION 2000 Inductively Coupled Plasma-Mass Spectrometer and a Syngistix™ Software-ES version 2.4 (PerkinElmer, USA). Instrumental settings were optimized in order to yield maximum sensitivity for platinum, with the scan mode Peak Hopping, Dwell time per AMU 50 ms and single MCA Channel. For quantitative determination, the most abundant isotopes of platinum and rhodium (used as internal standard) were measured at m/z 195 and 103, respectively. The assay method was evaluated with four kinds of matrix including blood, plasma, ultrafiltrate plasma, and cell lysate. Matrix effects were acceptable with non-spectral interferences. The standard curve was linear over a range 0.1–100 ng/ml. The correlation coefficient of the calibration curve by least-squares linear regression analysis was > 0.999. The lower limit of quantification of the present assay was 0.1 ng/ml. The precision (percentage coefficient of variation; % CV) of the assay method in different biological matrix was 10.8–12.9, 7.2–9.1, 5.2–6.9 and 4.0–5.8% at 0.1, 0.2, 10, and 80 ng/ml, respectively. And the accuracy of the method ranged from 91.5 to 102.8%.

#### Sample Treatment and Analysis for Intact Platinum-Based Drug Determination by UPLC-MS/MS

The biological samples were pretreatmented by precipitation with acetonitrile. To each 50 µl sample volume, 200 µl of acetonitrile containing IS (25 ng/ml) was added. The samples were then centrifuged for 10 min at 15,000 rpm, after which 5 µl of the supernatant was injected into the UPLC column for analysis of intact drug.

The determination of intact cisplatin, carboplatin, and oxaliplatin was performed with a UPLC-MS/MS system. The ACQUITY UPLC I class system consisted of an autosampler, pump, column oven, and controller (waters, MA, USA). Chromatographic separation was performed on an ACQUITY UPLC BEH Amide column (Part NO:186004800, 1.7 µm, 2.1 × 50mm, Waters, MA, USA) and the pre-column is ACQUITY UPLC BEH C18 (1.7µm, Waters, MA, USA). The binary mobile phase consisted of mobile phase B: acetonitrile and mobile phase A: water containing 5 mm ammonium acetate. The injection volume was 5 µl and the column temperature was 40°C. The gradient program was as follows: 0–1 min, 95% phase B; 1–2 min, 95–70% phase B; 2–4 min,70% phase B; 4–5 min, 70–95% phase B; 5–7min, 95% phase B. The total run time is 7.0 min and the flow rate was set at 0.6 ml/min.

The UPLC system was interfaced with a Xevo TQ-XS Mass Spectrometer with MassLynx V4.2 software package (Waters, MA, USA). The mass spectrometer was operated using Turbo Spray source in positive multiple-reaction monitoring-mode (MRM) for intact drug determination. Quantitation was performed using transitions of m/z 318.1 → 265.0 for cisplatin, m/z 372.3 → 294.1 for carboplatin, m/z 397.1→ 305.4 for oxaliplatin, and m/z 376.0 → 298.0 for internal standard (IS). The optimal parameters for MS/MS conditions are as follows: capillary 2.4 kV; cone 25 V; desolvation temperature 450 °C.

The assay method was evaluated with four kinds of matrix including blood, plasma, ultrafiltrate plasma, and cell lysate. Matrix effects were all acceptable. The standard curves for cisplatin, carboplatin, and oxaliplatin in blood/plasma/ultrafiltrate samples were linear over a range 2 to 5,000 ng/ml (R^2^ > 0.99). While for cell lysate, calibration curves over the concentration range of 2–200 ng/ml had correlation coefficients exceeding 0.999, indicating a good linearity over this concentration range. The lower limit of quantification is 2 ng/ml for the determination of three intact platinum analogues in blood, plasma, ultrafiltrate plasma, and cell lysate. The precision and accuracy for three intact platinum-based drugs in blood/plasma/ultrafiltrate plasma were investigated by replicate analysis of six sets of samples at four concentration levels: 2, 5, 200, and 4,000 ng/ml. The precision and accuracy for the assay method in cell lysate were investigated at four concentration levels of 2, 5, 20, and 160 ng/ml. The accuracy of the assay method varied between 91.7 and 106.8%, while the precision (% CV) was below 4.25%.

### The Study on Pharmacokinetics of Platinum-Based Anticancer Drugs at Animal Level

Male Sprague-Dawley rats (n = 18, 200 ± 20g) were provided by Shanghai Jiesijie Experimental Animal Co., Ltd (Shanghai, China) and certificate number was scxk (hu) 2013-0006. Animals were allowed to adapt to the housing environment for 1 week prior to study. The animals were fasted overnight (12 h) before the experimental and had free access to water throughout the experimental period. All animal studies were approved by the Animal Ethics Committee of China Pharmaceutical University.

Rats were randomly divided into three groups for intraperitoneal administration of cisplatin, carboplatin, and oxaliplatin respectively. The respective dosages for cisplatin, carboplatin, and oxaliplatin were 5, 6.19, and 6.63 mg/kg (all equivalent to Pt at 16.7 nmol/kg). Blood-sampling times were arranged as 0, 0.083, 0.5, 1, 2, 4, 8, 12, 24, 48, 72h post-dose for all three platinum-based anticancer drugs. When each time point reached, blood samples (about 800 µl) were collected from the fossa orbitalis. 150 µl of the blood was spiked into another 1.5 ml tube as the blood sample. The rest of the blood was centrifuged immediately at 8,000 rpm for 10 min and the supernatant plasma was harvested. 50 µl of the supernatant plasma was spiked into 1.5 ml for determining total platinum in plasma sample and 50 µl of the supernatant plasma was spiked into 1.5 ml tube for determining intact platinum-based drug in plasma. The remaining plasma was used to prepare ultrafiltrate plasma with Amicon^®^ Ultra 10K Centrifugal Filter Devices, centrifuged at 14,000 g for 20 min. All the samples were stored at -80 °C until analysis.

### The Study on Cellular Pharmacokinetics of Platinum-Based Anticancer Drugs at Cell Level

We chose the third-generation platinum-based drug oxaliplatin as the object to explore its cellular pharmacokinetics in two target cell lines (colon cancer HCT-116/LOVO cells).

First, we evaluated the uptake of oxaliplatin in colon cancer cells. For the time dependent study, HCT-116/LOVO monolayers were treated with 200 µm oxaliplatin-containing medium in a volume of 4 ml and then incubated for different times of 15, 30, 45, 60, 90, and 120 min. For the concentration dependent study, HCT-116/LOVO monolayers were treated with 4 ml oxaliplatin-containing medium at concentrations of 20, 50, 100, 200, and 500 µm for 30 min incubation. For the temperature dependent study, the cell monolayers were treated with 4 ml drug-containing medium at 200 µm for 30 min incubation respectively at 4 °C and 37 °C.

Second, the elimination of oxaliplatin in HCT-116/LOVO cells was investigated. HCT-116/LOVO monolayers were treated with 200 µm oxaliplatin-containing medium in a volume of 4 ml for 30 min. Then the drug-containing medium was discarded and the cell monolayers were washed three times with cold HBSS. After that, fresh blank medium was added to the monolayers with incubation for different times of 5, 15, 30, 45, 60, 120 min.

Third, we investigated the subcellular distribution of oxaliplatin in HCT-116/LOVO cells. We treated the cell monolayers in the same way as the uptake study above.

Finally, all the monolayers of cells treated with drug-containing medium were washed three times with cold HBSS. Then cells were harvested by 0.25% trypsin digestion and the resulting cell suspension was centrifuged at 1,000 rpm for 5 min. For the uptake and elimination studies, pellets were then diluted in appropriate volume of water and repeatedly freeze-thawed three times to obtain drug-containing cell samples. For the subcellular distribution study, the pellets were further treated according to the manufacturer’s protocol of mitochondrial/nuclear separation kit (KeyGEN BioTECH, Nanjing, China) with differential centrifugation method to prepare the isolated cytosol, nucleus, and mitochondria samples. The cytosol, nucleus, and mitochondria were dissolved in the corresponding buffer salt from the instruction. An aliquot of 20 μl of each cellular or subcellular sample was removed for protein determination using BCA protein assay kit according to the manufacturer’s instructions (micro-well plate protocol).

### Pharmacokinetic Parameters and Statistical Analysis

The pharmacokinetic parameters were calculated using the software WinNonlion Version 6.4 by non-compartmental method. Statistical significance was tested by Student’s *t* -test. *P* < 0.05 was considered statistically significant, while *P* < 0.01 was considered highly significant.

## Results

### Pharmacokinetics of Three Platinum-Based Anticancer Drugs in Rats

In order to explore and compare the pharmacokinetics of cisplatin, carboplatin, and oxaliplatin comprehensively, both total platinum and intact platinum-based anticancer drugs in all of samples (blood, plasma, or plasma ultrafiltrate) were determined by ICP-MS and UPLC-MS/MS methods.

**Figure 2:**
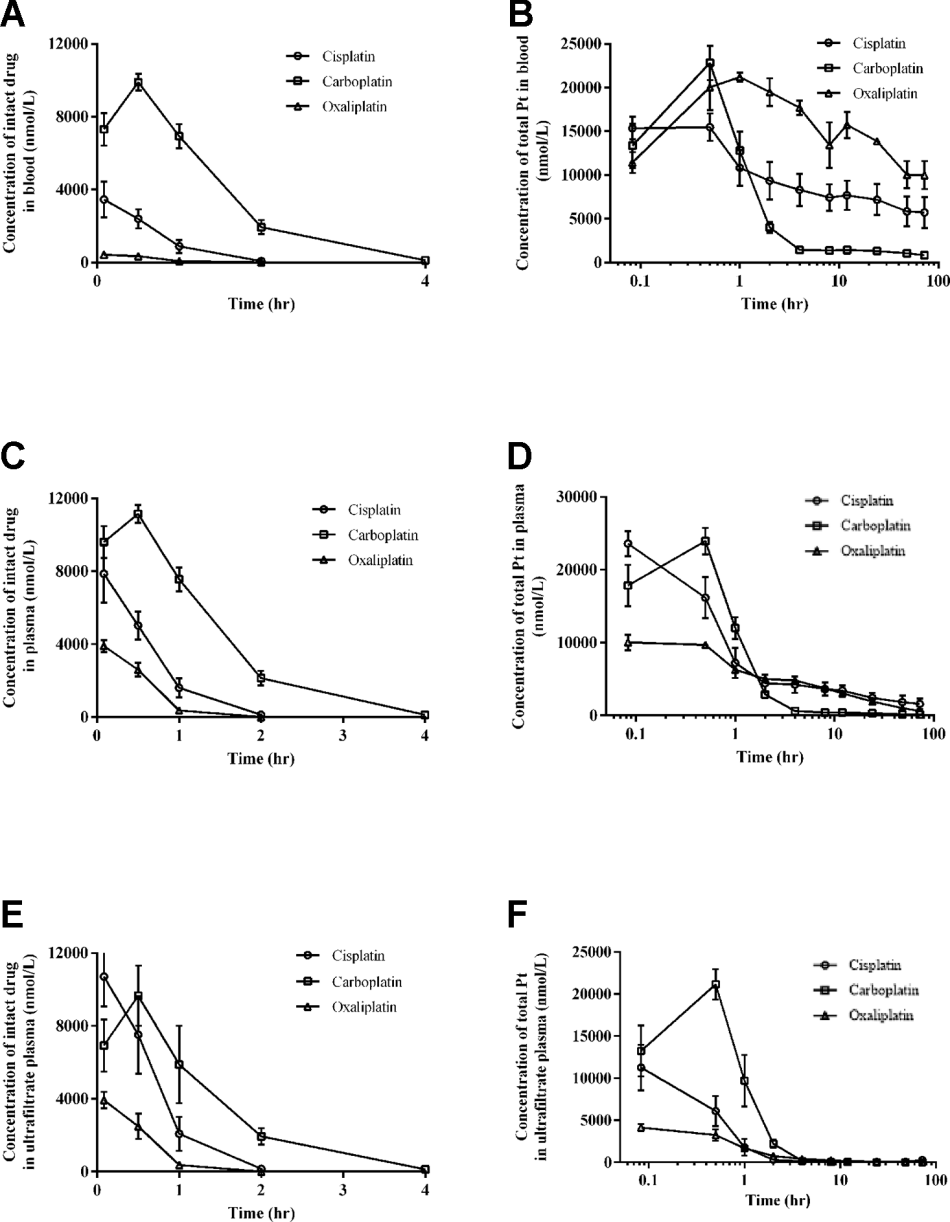
Intact drug and total platinum concentration-time profiles in blood **(A** and **B)**, plasma **(C** and **D)**, and ultrafiltrate plasma **(E** and **F)** after intraperitoneal injection of equal moles of three platinum-based drugs to rats (equivalent to platinum at 16.7 nmol/kg). All data are presented as mean ± sd (n = 6).

The intact drug and total Pt concentrations in blood for three platinum analogues over time are presented in **Figures 2A**, **B**. The concentrations of intact platinum analogues and total Pt in plasma over time are shown in **Figures 2C**, **D**. **Figures 2E**, **F** present the concentrations of intact drugs and total Pt in ultrafiltrate plasma over time. The pharmacokinetic parameters for three drugs based on intact drugs and total platinum are respectively summarized in **Tables 1** and **2**.

**Table 1:** The pharmacokinetic parameters of intact platinum-based drugs in in blood, plasma and ultrafiltrate plasma after intraperitoneal injection of equal moles of three platinum-based drugs to rats (16.7 nmol/kg).

Samples	ID	T_1/2_	T_max_	C_max_	AUC_0-72h_	Vz	Cl
	Intact drug	hr	hr	mmol/L	hr*mmol/L	L/kg	L/hr/kg
Blood	cisplatin	0.30 ± 0.02	0.08 ± 0.00	3.46 ± 0.97	2.66 ± 0.53	0.0028 ± 0.0006	0.0064 ± 0.0012
	carboplatin	2.04 ± 0.13	0.50 ± 0.00	9.90 ± 0.45	14.91 ± 1.04	0.0033 ± 0.0004	0.0011 ± 0.0001
	oxaliplatin	0.55 ± 0.04	0.08 ± 0.00	0.42 ± 0.06	0.34 ± 0.04	0.0408 ± 0.0058	0.0490 ± 0.0066
							
Plasma	cisplatin	0.28 ± 0.03	0.08 ± 0.00	7.86 ± 1.59	5.53 ± 0.75	0.0012 ± 0.0002	0.0030 ± 0.0004
	carboplatin	1.95 ± 0.12	0.50 ± 0.00	11.15 ± 0.49	16.85 ± 1.14	0.0028 ± 0.0003	0.0001 ± 0.0000
	oxaliplatin	0.18 ± 0.00	0.08 ± 0.00	3.90 ± 0.34	2.44 ± 0.23	0.0018 ± 0.0001	0.0069 ± 0.0006
							
	cisplatin	0.26 ± 0.02	0.17 ± 0.18	11.45 ± 1.46	8.33 ± 0.70	0.0008 ± 0.0001	0.0020 ± 0.0002
Ultrafiltrate plasma	carboplatin	1.52 ± 0.49	0.42 ± 0.19	10.12 ± 0.75	13.92 ± 2.48	0.0027 ± 0.0009	0.0012 ± 0.0002
	oxaliplatin	0.18 ± 0.01	0.08 ± 0.00	3.93 ± 0.45	2.40 ± 0.47	0.0019 ± 0.0006	0.0072 ± 0.0017

^*^All data are presented as mean ± sd (n = 6).

**Table 2:** The pharmacokinetic parameters of total platinum in blood, plasma and ultrafiltrate plasma after intraperitoneal injection of equal moles of three platinum-based drugs to rats (16.7 nmol/kg).

Samples	ID	T_1/2_	T_max_	C_max_	AUC_0-72h_	V_z_	Cl
	Total Pt	hr	hr	mmol/L	hr*mmol/L	L/kg	L/hr/kg
Blood	Pt from cisplatin	104.3 ± 12.1	0.33 ± 0.23	16.1 ± 1.2	437.1 ± 60.5	0.002 ± 0.000	0.00001 ± 0.00000
	Pt from carboplatin	80.7 ± 23.9	0.50 ± 0.00	22.8 ± 1.9	116.1 ± 10.1	0.009 ± 0.001	0.00008 ± 0.00002
	Pt from oxaliplatin	72.6 ± 14.2	0.88 ± 0.25	21.7 ± 1.2	900.3 ± 46.2	0.001 ± 0.000	0.00001 ± 0.00000
							
Plasma	Pt from cisplatin	40.4 ± 2.4	0.08 ± 0.00	23.6 ± 1.7	160.1 ± 17.5	0.004 ± 0.000	0.00007 ± 0.00001
	Pt from carboplatin	30.4 ± 3.6	0.50 ± 0.00	23.9 ± 1.8	46.4 ± 3.2	0.014 ± 0.002	0.00033 ± 0.00003
	Pt from oxaliplatin	28.4 ± 2.0	0.22 ± 0.24	10.3 ± 0.7	139.0 ± 5.9	0.004 ± 0.000	0.00010 ± 0.00000
							
Ultrafiltrate plasma	Pt from cisplatin	38.1 ± 10.3	0.08 ± 0.00	11.3 ± 2.7	14.0 ± 3.6	0.025 ± 0.011	0.00078 ± 0.00050
	Pt from carboplatin	16.4 ± 2.5	0.50 ± 0.00	21.2 ± 1.8	25.2 ± 2.7	0.016 ± 0.002	0.00067 ± 0.00007
	Pt from oxaliplatin	17.9 ± 3.7	0.08 ± 0.00	4.1 ± 0.4	11.3 ± 1.9	0.038 ± 0.001	0.00140 ± 0.00022

^*^All data are presented as mean ± sd (n = 6).

#### Pharmacokinetics of Three Platinum-Based Anticancer Drugs in SD Rats Based on Quantification of Intact Drugs

For intact platinum analogues in blood, the mean AUC_0-72_ values were 2.66 for cisplatin, 14.91 for carboplatin, and 0.34 hr*mmol/L for oxaliplatin. Mean Cl values were 0.0064 for cisplatin, 0.0011 for carboplatin, and 0.0490 L/hr/kg for oxaliplatin. Mean V_z_ values were 0.0028 for cisplatin, 0.0033 for carboplatin, and 0.0408 L/kg for oxaliplatin.

For intact platinum analogues in plasma, the mean AUC_0-72_ values were 5.53 for cisplatin, 16.85 for carboplatin, and 2.44 hr*mmol/L for oxaliplatin. Mean Cl values were 0.0030 for cisplatin, 0.0001 for carboplatin, and 0.0069 L/hr/kg for oxaliplatin. Mean V_z_ values were 0.0012 for cisplatin, 0.0028 for carboplatin, and 0.0018 L/kg for oxaliplatin.

For intact platinum in ultrafiltrate plasma, the mean AUC_0-72_ values were 8.33 for cisplatin, 13.92 for carboplatin, and 2.40 hr*mmol/L for oxaliplatin. Mean Cl values were 0.0020 for cisplatin, 0.0012 for carboplatin, and 0.0072L/hr/kg for oxaliplatin. Mean V_z_ values were 0.0008 for cisplatin, 0.0027 for carboplatin, and 0.0019 L/kg for oxaliplatin.

Intact oxaliplatin and cisplatin have a fast elimination, with a complete elimination of 2 h. Compared to cisplatin and oxaliplatin, carboplatin has a longer complete elimination of 12 h.

#### Pharmacokinetics of Three Platinum-Based Anticancer Drugs in SD Rats Based on Quantification of Total Platinum

For total platinum, the pharmacokinetic behaviors of three platinum analogues are completely different from those based on intact drugs.

For total Pt in blood, the mean AUC_0-72h_ values were 437.1 for cisplatin, 116.1 for carboplatin, and 900.3 hr*mmol/L for oxaliplatin. Mean Cl values were 0.00001 for cisplatin, 0.00008 for carboplatin, and 0.00001 L/hr/kg for oxaliplatin. Mean V_z_ values were 0.002 for cisplatin, 0.009 for carboplatin, and 0.001 L/kg for oxaliplatin.

For the total Pt in plasma, the mean AUC_0-72h_ values were 160.1 for cisplatin, 46.4 for carboplatin, and 139.0 hr*mmol/L for oxaliplatin. Mean Cl values were 0.00007 for cisplatin, 0.00033 for carboplatin, and 0.00010 L/hr/kg for oxaliplatin. Mean V_z_ values were 0.004 for cisplatin, 0.014 for carboplatin, and 0.004 L/kg for oxaliplatin.

For the total Pt in ultrafiltrate plasma, the mean AUC_0-72h_ values were 14.0 for cisplatin, 25.2 for carboplatin, and 11.3 hr*mmol/L for oxaliplatin. Mean Cl values were 0.00078 for cisplatin, 0.00067 for carboplatin, and 0.00140 L/hr/kg for oxaliplatin. Mean V_z_ values were 0.025 for cisplatin, 0.016 for carboplatin, and 0.038 L/kg for oxaliplatin.

Compared to cisplatin and oxaliplatin, the clearance and V_z_ of platinum were relatively high in plasma and blood for carboplatin but relatively low in ultrafiltrate plasma. Moreover, the AUC_0-72h_ of platinum from carboplatin was the lowest in plasma and blood, but the highest in ultrafiltrate plasma among the three platinum analogues.

### Cellular Pharmacokinetics of Oxaliplatin in Colon Cancer Cells

#### The Uptake of Oxaliplatin in HCT-116/LOVO Cell Lines

**Figure 3:**
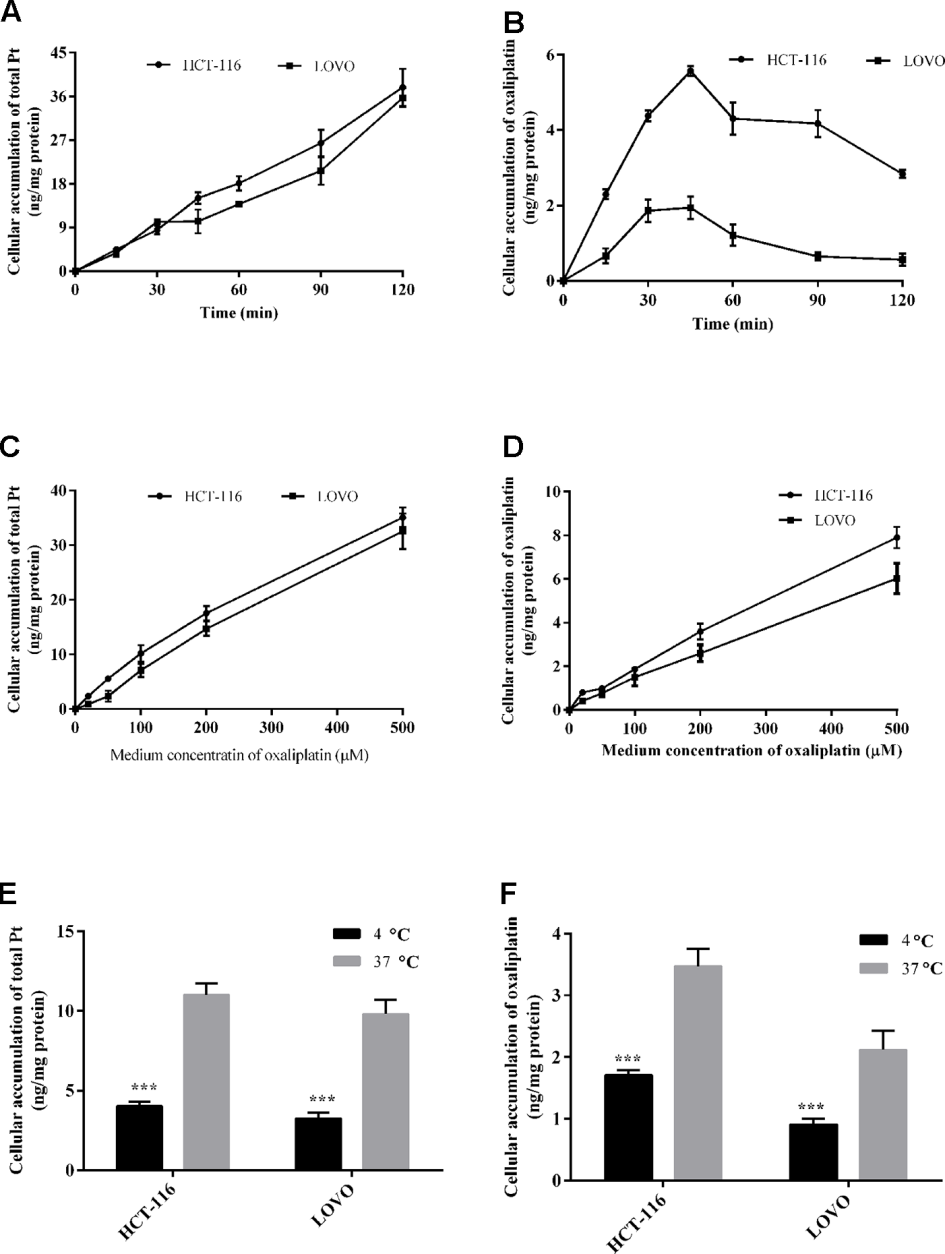
Cellular total platinum **(A)** and intact oxaliplatin **(B)** over time with 200 μm oxaliplatin exposure to HCT-116 and LOVO cell lines. Uptake of total platinum **(C)** and intact oxaliplatin **(D)** in HCT-116 and LOVO cell lines at different medium concentrations of oxaliplatin (20–500 μM) for 30 min incubation. The cellular total platinum **(E)** and oxaliplatin **(F)** uptake as a function of treatment temperature with 200 μm oxaliplatin exposure to HCT-116 and LOVO cell lines for 30 min. All data are presented as mean ± sd (n = 3). The uptake difference between two groups at 4^◦^C and 37^◦^C was highly significant (***P < 0.001).

In this study, we evaluated the effects of time, concentration, and temperature on the uptake of oxaliplatin in colon cancer cells (**Figure 3**). Both oxaliplatin and total platinum in all of the samples were determined by UPLC-MS/MS and ICP-MS methods. The presented concentrations of oxaliplatin or total platinum were all normalized to 1 mg cellular protein.

For the time dependent study, **Figures 3A, B** show the observed total platinum and intact oxaliplatin concentrations (ng/mg protein) for the HCT-116/LOVO cell lines exposed to 200 µm oxaliplatin at different times. For the total platinum, the incorporation Pt in both HCT-116 and LOVO cells increased within increasing incubation time. While for the intact oxaliplatin, the cellular accumulation of oxaliplatin in two colon cancer cell lines both increased over incubation time at the beginning of drug treatment (0–45 min), then gradually decreased (45–120 min), which is different from the trend of intact oxaliplatin.

For the concentration dependent study, the cellular accumulation of platinum and oxaliplatin in HCT-116/LOVO cells after exposure to oxaliplatin at different concentrations for 30 min is shown in **Figures 3C, D**. As shown in the figure, whether the concentration of total platinum or intact oxaliplatin in two colon cancer cell lines both increased with increasing medium concentration of oxaliplatin.

For the temperature dependent study, the Pt or oxaliplatin incorporation results for HCT-116 and LOVO cells after exposure dose of oxaliplatin at different temperatures are provided in **Figures 3E, F**. The values for cellular accumulation of platinum or oxaliplatin in HCT-116/LOVO cells are significant higher at 37°C than that at 4°C.

#### The Elimination of Oxaliplatin in HCT-116/LOVO Cell Lines

To evaluate the elimination behavior of oxaliplatin in colon cancer cells, both oxaliplatin and total platinum of samples are determined by UPLC-MS/MS and ICP-MS method. The results are all normalized to cell protein.

**Figures 3C, D** show the total platinum and oxaliplatin concentration results for HCT-116/LOVO cells after exposed to blank medium at different times in the back of exposure to 200 µm oxaliplatin for 30 min. The concentration of cellular intact oxaliplatin shows a very similar behavior in both HCT-116 cell line and LOVO cell line, with a rapid decrease and complete elimination in 1 h. However, the cellular platinum in HCT-116/LOVO cells just has a little decrease in 2 h. There is an obvious difference in the behaviors between oxaliplatin and total platinum.

#### The Subcellular Distribution of Oxaliplatin in HCT-116/LOVO Cells

All the prepared subcellular samples for cytosol, nucleus, and mitochondria are determined by ICP-MS method. The Pt concentration results in subcellular organelles of HCT-116/LOVO cells within 2 h are shown in **Figure 5**. Whether in HCT-116 cells or LOVO cells, the subcellular accumulation of Pt in organelles increased with the increasing incubation time. In addition, the concentration of Pt in cytosol is more than that in mitochondria, followed by the concentration of Pt in nucleus.

## Discussions

The pharmacokinetics of three platinum analogues are systematically investigated and compared at animal level, based on quantification of total platinum and intact drugs in blood, plasma, and plasma ultrafiltrate. After administration of equimolar amount of three platinum analogues, carboplatin has the lowest total platinum exposure in blood and plasma compared to cisplatin and oxaliplatin (**Figures 2B**, **D**). However, there is a different distribution trend in ultrafiltrate plasma, with carboplatin the highest exposure (**Figure 2F**). These results indicate that carboplatin has the highest distribution in ultrafiltrate plasma compared to cisplatin and oxaliplatin, which may be caused by its stronger affinity for low molecular weight nucleophiles (Jacobs et al., 2005). However, there is a significant difference between the intact drug and total platinum distribution trends (**Figures 2A**, **C**, **E**). All of the intact drugs have fast elimination, which is mainly caused by their rapid biotransformation due to their strong electrophilicity (Bandu et al., 2015). Among the three platinum analogues, carboplatin has a relatively long complete elimination time (12 h) compared to cisplatin (2 h) and oxaliplatin (2 h). The ultrafiltrate platinum contains the intact drug and the low molecular weight platinum metabolites, which are generally known to be the main active ingredients (Dabrowiak et al., 2012). From the study, we can know that carboplatin has the highest proportion of platinum distribution in ultrafiltrate plasma and also has the longest intact drug elimination time in blood, plasma, and ultrafiltrate plasma among the three platinum analogues, which may be one of the main reasons for its potent blood marrow toxicity (Milic et al., 2019). The significantly different pharmacokinetic behaviors between intact drug and total platinum indicate that evaluation on pharmacokinetics of platinum-based anticancer drugs with only total platinum or only intact drugs has certain limitations. A multi-angle evaluation of platinum-based anticancer drugs in blood, plasma, and ultrafiltrate plasma with simultaneous quantification of total platinum and intact platinum analogues can provide us more comprehensive and valuable information for the study of platinum-based anticancer drugs.

**Figure 4:**
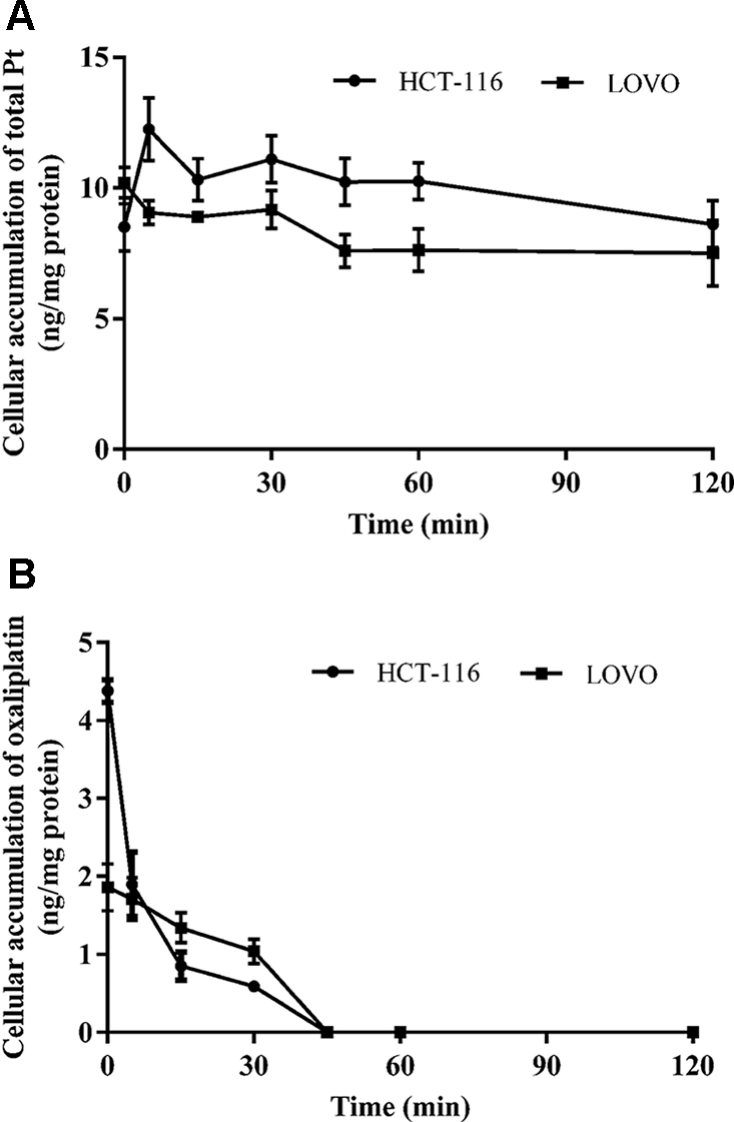
The time course of total platinum **(A)** and oxaliplatin **(B)** in HCT-116 and LOVO cell lines after incubation with 200 μm drug-containing medium for 30 min and then replaced by fresh medium without drug. All data are presented as mean ± sd (n = 3).

Although the uptake of platinum-based drugs in cells has been reported, most of the previous studies all focused on determination of only total Pt or only intact drugs in cells (Ghezzi et al., 2004; Gabano et al., 2008; Qin et al., 2018). However, simultaneous assessment of both intact drug and total platinum in cells is of great value for the comprehensive understanding of pharmacokinetic behaviors of platinum-based anticancer drugs at the cell level. In addition, the elimination and subcellular distribution of platinum-based drugs in target cancer cells have not been reported though the subcellular distribution of platinum-based drugs in normal tissues and cells has already been investigated by (Esteban-Fernandez et al., 2008). Even though cisplatin, carboplatin, and oxaliplatin are all currently used in clinic, oxaliplatin as the third-generation platinum-based drug has the most specific indication (Kong et al., 2016) (good colon cancer treatment effect but not for other cancers). Therefore, we chose oxaliplatin as the research subject to investigate its cellular pharmacokinetics in target cells. Among kinds of colon cancer cell lines, HCT-116 and LOVO are respectively the typical primary and metastatic colon cancer cell lines, which are used as the two target cell models in our study. The pharmacokinetics of oxaliplatin in target cells was systematically evaluated at cellular and subcellular levels, with quantification of both intact oxaliplatin and total platinum. Some results show a significant difference in the behaviors of intact oxaliplatin and total platinum in cells, especially in the time dependent study and elimination study. **Figure 3B** shows a first increasing trend of cellular oxaliplatin, but then a decreasing trend. However, the cellular Pt just has the increasing trend within 2 h (**Figure 3A**). When the uptake of oxaliplatin by cells started, the absorption and elimination of oxaliplatin occurred simultaneously in cells, with absorption oriented at the beginning and then elimination oriented (Sprowl et al., 2013; Wagner et al., 2016). Although part of oxaliplatin converted into other platinum-containing products in cells, which was still within cells so as to make an increasing total platinum in cells. Taken together with the results of elimination study (**Figures 4A**, **B**), the rapid decrease of cellular oxaliplatin, but just little decrease of total platinum in cells can also be explained. These results indicated that the efflux of the platinum-containing products from oxaliplatin biotransformation is little. Most of the platinum-containing products are biomacromolecular conjugates (Messori and Merlino, 2016), which are not easily excreted from cells. The result showing cellular oxaliplatin/Pt increased linearly with concentration (**Figures 3C**, **D**) suggests a passive transport involved in the cellular uptake of oxaliplatin (Gabano et al., 2008). On the other hand, the uptake of oxaliplatin indicated the temperature dependent property (**Figures 3E**, **F**) (Shen et al., 2013; Cui et al., 2017). Even though the cellular oxaliplatin and total platinum have similar trends both in the concentration dependent experiment and temperature dependent experiment, the values for the concentrations of oxaliplatin are much lower than that for total platinum, which is consistent with the rapid conversion of oxaliplatin in cells.

**Figure 5:**
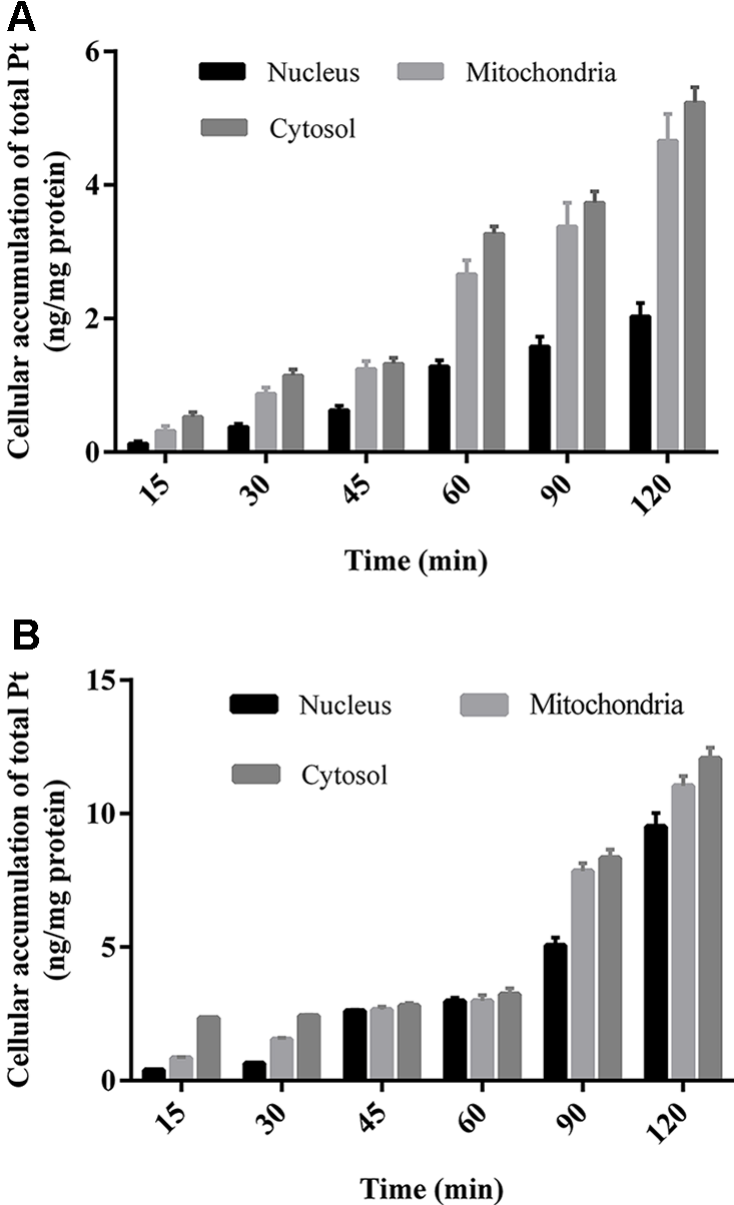
The concentration of Pt in different subcellular organelles of HCT-116 cells **(A)**/LOVO cells **(B)** at different incubation time treated with 200 μm drug-containing medium. All data were expressed as (ng Pt/mg protein of subcellular organelles) and presented as mean ± sd (n = 3).

The subcellular distribution study shows that the accumulation of platinum in mitochondria is higher than that in nucleus (**Figures 5A, B**). It is generally believed that platinum-based anticancer drugs have the antitumor effect by interacting with DNA in nucleus (Corte-Rodriguez et al., 2015). The enrichment of platinum in mitochondria may affect the respiratory chain and energy metabolism, which may further lead to cell death (Kalyanaraman et al., 2018). It is speculated mitochondria may be another potential target for oxaliplatin in antitumor effect, which remains to be confirmed by further experiments.

Systematic assessment of the pharmacokinetics for platinum analogues with quantification of total platinum and intact drugs at both animal and target cell levels can help us understand the pharmacokinetics of platinum-based anticancer drugs comprehensively, which can further provide a scientific basis for the study of their efficacy and toxicity. The research also provide an important reference for the pharmacokinetic study of other novel platinum-based anticancer drugs.

## Data Availability Statement

The datasets generated for this study are available on request to the corresponding author.

## Ethics Statement

The animal study was reviewed and approved by the Animal Ethics Committee of China Pharmaceutical University.

## Author Contributions

ZQ, GR, JY, etc. are responsible for the operation of the experiment, data processing and writing. HC, YL, NL, YZ, etc. are responsible for interpretation of data, revising it critically and writing guidance of the experimental program. XC, DZ, etc. are responsible for the design of the work, program development and revising it critically for important intellectual content.

## Funding

This work was supported by the National Natural Science Foundation of China (No. 81503148, No. 81473272).

## Conflict of Interest

The authors declare that the research was conducted in the absence of any commercial or financial relationships that could be construed as a potential conflict of interest.
